# Evaluation of the osteogenic potential of demineralized and decellularized bovine bone granules following implantation in rat calvaria critical-size defect model

**DOI:** 10.1371/journal.pone.0294291

**Published:** 2023-12-21

**Authors:** Ali Al Qabbani, K. G. Aghila Rani, Sausan AlKawas, Suzina Sheikh Abdul Hamid, Abdullah Yap Abdullah, A. R. Samsudin, Ahmad Azlina

**Affiliations:** 1 Oral and Craniofacial Health Sciences Department, College of Dental Medicine, University of Sharjah, Sharjah, United Arab Emirates; 2 School of Dental Sciences, Universiti Sains Malaysia, Kota Bharu, Malaysia; 3 Research Institute for Medical and Health Sciences, University of Sharjah, Sharjah, United Arab Emirates; 4 Tissue Bank, School of Medical Sciences, Universiti Sains Malaysia, Kota Bharu, Malaysia; Ohio State University, UNITED STATES

## Abstract

The aim of this study was to compare the ability of demineralized (DMB) and decellularized (DCC) bovine bone granules to support bone regeneration in rat calvaria critical-size defects. DMB and DCC were prepared using a previously published method. The granule size used ranged between 500 and 750 μm. A total of forty-eight Sprague-Dawley rats were divided into two groups (n = 24). A pair of 5 mm diameter defects were created on the calvaria of the rats in the right and left parietal bone in both groups. Group A animals received DMB granules and Group B received DCC granules in the right parietal defect side while the left parietal untreated defect acted as sham surgery for both groups. Four animals per group were euthanized in a CO_2_ chamber at day 7, 14 and 21 post-surgery and the calvaria implantation site biopsy harvested was subjected to osteogenic gene expression analysis. Another four animals per group were euthanized at days 15, 30 and 60 post surgery and the calvaria implantation site biopsy harvested was subjected to histological, immunohistochemistry, RAMAN spectroscopy and Micro-CT analysis at the mentioned time points. Statistical analysis was conducted using t-tests and ANOVA. Histomorphometry showed significantly higher new bone formation in the DCC sites (p<0.05) compared to DMB. Both DMB and DCC implantation sites showed distinct staining for osteocalcin and osteopontin proteins compared to their respective sham sites. By day 21 after implantation, DCC sites demonstrated significantly elevated mRNA levels of osteonectin (p<0.001), osteopontin (p<0.001), osteocalcin (p<0.0001), ALP (p<0.01), and BMP-2 (p<0.001) compared to DMB. However, VEGF expression showed no significant differences at this time point between the two groups. Micro-CT analysis also showed enhanced defect closure and higher bone density in DCC implanted sites while RAMAN spectra demonstrated increased abundance of collagen and bone minerals, especially, PO_4_^3-^ ions than DMB. In conclusion, both DMB and DCC granules demonstrated favorable osteogenic potential in critical-sized defects, with DCC exhibited superior osteoconductive, osteoinductive and osteogenesis properties.

## Introduction

Bone graft substitutes have been employed in clinical practice for hundreds of years, beginning from the earliest documented utilization of bone grafts in 1682 [1]. In recent times, their application in dentistry has witnessed a significant rise, primarily due to advancements in dental implantology and the increasing demand for reconstructing craniofacial congenital and acquired bone defects [2, 3]. Bone grafting is required for one in every four dental implants, as a commonly routine practice in dentistry highlighting the high demand in general practice alone [4].

In clinical practice, bone regeneration is a crucial aspect of treating defects resulting from trauma, congenital issues, and tumor removal. Although several clinical methods are available to tackle these defects, the non-union defect, which is the incomplete closure of the defect, remains a significant challenge [5]. To address this challenge, studies utilizing animal models such as rabbits, mice, dogs, pigs, sheep, and rats have provided valuable insights and knowledge to test optimal bioscaffolds for bone tissue engineering applications [6]. Orthotopic rodent models provide the most clinically relevant experimental system for evaluating biomaterials or strategies for non-union applications [6]. An example of this is the rat calvaria defect model, which involves repairing bone sites in their original location and produces physiologically applicable results that are superior to those obtained by inducing bone growth in ectopic sites [7]. This model was chosen due to its widespread recognition in the literature as one of the most employed preclinical models for assessing the regenerative capabilities of various biomaterials for bone substitutes [8]. Rodent calvaria models allow the possibility of precise comparison of grafted substances; in addition, there is no requirement for internal or external fixation of the bone grafts to be tested because the dura and the scalp provide a suitable foundation for implanted bone substitutes [9]. Before progressing to larger animals for potential application in the human craniofacial complex, the rat calvaria defect model can be employed to evaluate bone healing and test various biomaterials or tissue engineering constructs.

Previous studies have demonstrated the potential benefits of using biomaterials such as hydroxyapatite (HAP), tricalcium phosphates, and nano-hydroxyapatite (nHAP) in different configurations as bone substitute materials, which exhibit high biocompatibility in calvaria defect models [7]. Although these composite forms are easy to handle and effectively prevented macrophage phagocytosis at the implant sites, new bone formation was observed to be diminishing by 8 weeks after implantation due to the rapid bio-absorption of the material [7, 10].

The two main types of natural bone grafts used in dentistry are demineralized and deproteinized or decellularized bone grafts. While demineralized bone grafts have gained popularity over the last five decades and have proven effective in dental practice, decellularized bone grafts seem to exhibit less long-term immunogenicity and chronic inflammatory reactions. Recently more interest have been shown in the development of decellularized bovine bone grafts that claimed superior biocompatibility and osteogenic enhancement for potential clinical applications [11, 12].

A recent study from our group reported the development of a novel decellularization technique for the production of biocompatible bovine cancellous scaffolds for bone regeneration applications [13]. With minimal damage to the extra cellular matrix (ECM), our technique created a decellularized (DCC) scaffold with superior biocompatibility properties that is capable of inducing osteogenesis *in vitro* through the regenerative pathways of osteoconduction, osteoinduction, and osteogenesis.

The aim of this study was to investigate the efficacy of decellularized cancellous bovine bone granules as a potential bone substitute and compared it to demineralized cancellous bovine bone granules in healing of rat calvaria critical-size defect model.

## Material and methods

### Animals

Animal experiments were approved and conducted in agreement with the Animal Care and Use Committee, University of Sharjah, Sharjah, UAE (ACUC-18-10-22-01) and University Sains Malaysia, Malaysia (USM/IACUC/2021/127/1135). Eight weeks-old male Sprague-Dawley rats (Janvier labs; Rodent research models, France), weighing 250-350g were used in the experiments. The rats were randomly divided into two groups, each consisting of three rats and allowed 1 week to adapt to their environment before the treatments. Animals were housed in single cages with a labelling card attached to the cage, at a temperature of 22 ± 2°C with a 12-h dark/light cycle and ad libitum access to food and water. Animals were regularly monitored for signs of pain or infection, food intake and activity during the entire experimental period. An assigned veterinarian in the animal facility along with the research group performed their daily round and inspections to the animals. This report was prepared following the ARRIVE guidelines for reporting animal research [14].

**Fig 1:**
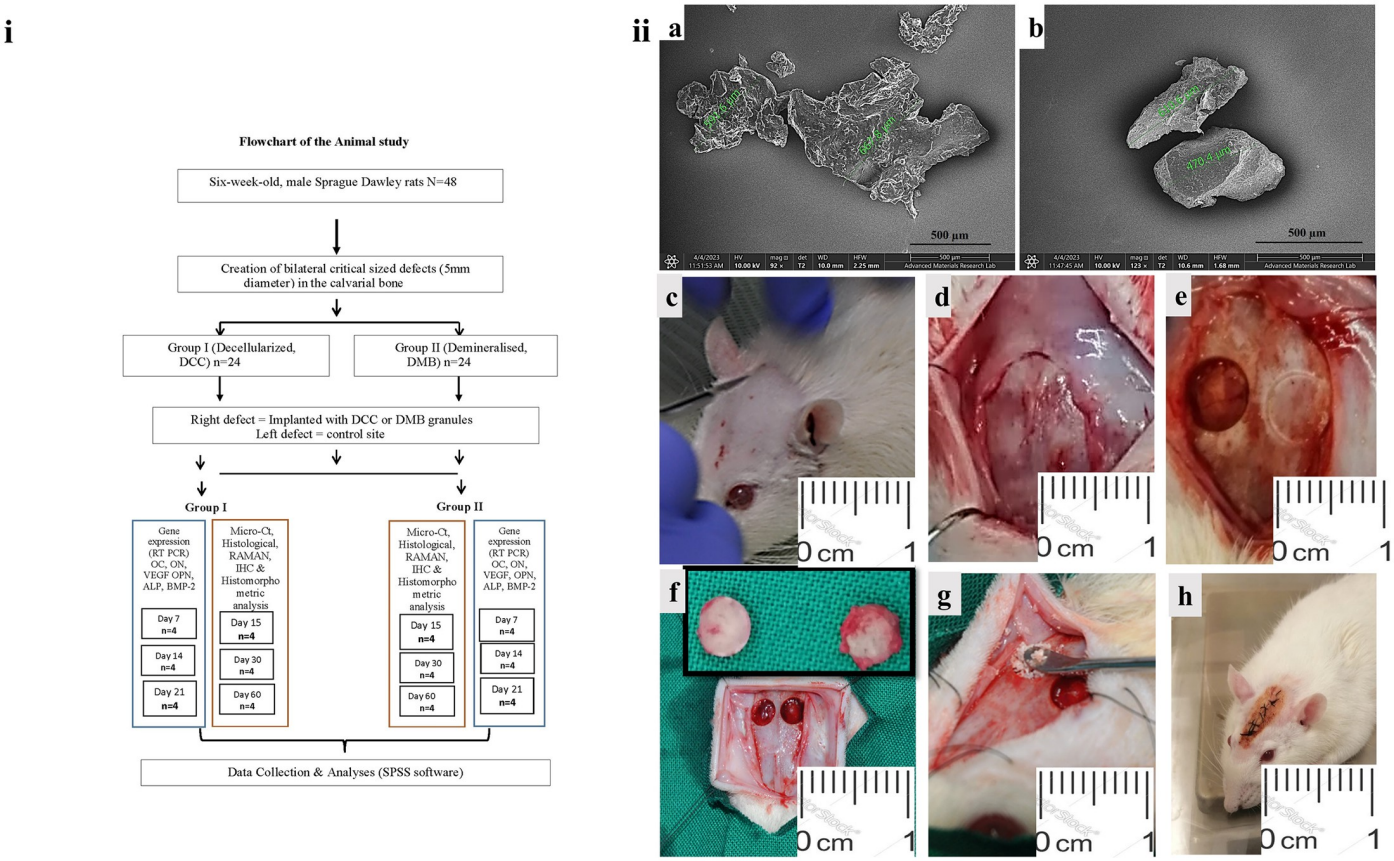
(i) Flowchart of the animal study. (ii) SEM analysis of bovine bone granules and implantation surgery procedure. The (a) DMB and (b) DCC granules ranged in size from 500–750μm. (c) Experimental implantation surgery of DMB and DCC bovine bone granules begins with scalp preparation and incision; (d) Full-thickness scalp flap elevation and reflection exposing the calvaria bone; (e) Creation of two calvaria critical-sized defects, 5 mm in diameter on left and right side of parietal bone; (f) Demonstration of two identical calvaria critical-sized defects with the trephined bone plug shown in the inset; (g) Implantation of DMB or DCC bovine bone granules on the right parietal bone defect with a sham defect on the left parietal side; (h) Scalp flap replaced and sutured using silk sutures followed by application of antiseptic solution 10% betadine.

Based on previous studies, it was estimated that a minimum sample size of 10 animals/group was necessary to achieve 80% power to detect a mean difference of 30% in defect closure between experimental groups (defect closure: 30 ±15% vs. 60 ±30%) [15, 16]. A total of 48 male Sprague-Dawley rats, eight-week old and weighing 250g-350g ± 16 g were used in the present study. The animals were randomly divided into two main groups of 24 animals each (n = 24). One group of rats received DMB granules while the other received DCC granules. The rats were also randomly distributed to subgroups according to the experimental procedure and specific time points. (Fig 1(i)).

### Preparation of Cancellous bovine bone granules

Cancellous bovine bone granules were prepared from fresh femur bone harvested from 24-month-old calves. The cancellous bone was separated and cut into 10mm^3^ blocks. The bone blocks were divided into two groups and were processed into lyophilized demineralized bone (DMB) and lyophilized decellularized bone (DCC). The processing techniques were described in detail in our previous study [13].

Both the DMB and DCC blocks were crushed into granules using an IKA A10 S9 bone grinder (Staufen, Germany) and the size of the granules produced was in the range of between 500 μm and 750 μm (Fig 1(ii)a & 1(ii)b); as was determined using SEM (VEGA3 XM-TESCAN, Czech Republic). The cancellous bovine bone granules obtained were lyophilized and radio-sterilized at 25 kGy, and the final product produced are demineralized bovine bone granules (DMB) and decellularized bovine bone granules (DCC) that were used in the *in vivo* implantation experiments in this study.

Animals were anesthetized using a combination of xylazine (20–80mg/kg body weight) and ketamine (80–100mg/kg body weight). The rat calvariae were shaved and cleaned with betadine followed by a local injection of an anesthetic agent (0.2 mL, 2% lidocaine with 1:80,000 epinephrine). Full-thickness scalp flaps were then elevated exposing the calvaria, and two circular critical-size defects, 5mm in diameter were created in the parietal bone on each side of the mid-sagittal suture using a trephine dental bur connected to a slow-speed handpiece, and under sterile saline irrigation [17]. The circular bone plug was removed carefully without harming the dura. In Group A, 22 mg of DMB granules were implanted in the right calvaria defect using a sterile spoon scoop while the left defect was left as a sham surgery. In Group B, 22 mg of DCC granules was implanted in the right calvaria defect while the left defect was again left as a sham surgery.

The scalp flaps were replaced and closed with silk sutures (Softretch 4–0, GC). The surgical procedures are illustrated in Fig 1(ii)c–1(ii)h. All animals were housed in single cages and received a daily intramuscular injection of Bupresol at a dose of 20,000 U/kg for three consecutive postoperative days. Four animals per group were euthanized in a CO_2_ inhalation chamber at days 7, 14, and 21 post-surgery for gene expression studies while another four animals per group were euthanized at days 15, 30 and 60 for histological and immunohistochemistry, micro-CT analysis and Raman spectroscopy [18–20].

### Gene expression

For gene expression studies, total RNA was extracted from the tissue in the region of interest at the site of implantation and sham site using TRIzol. We identified and isolated the region of interest from the cranial area using a low-speed motor equipped with a disc bur. Subsequently, the sham and the treated sites were separated by means of the sagittal suture, with each region designated as either "sham" or "DMB/DCC implanted." After this step, tissue sections were subjected to homogenization and subsequently underwent the RNA extraction process. The obtained RNA was purified using a pure LINK RNA extraction kit (Invitrogen, USA). RNAs were transcribed to cDNA using SuperScript^™^ II reverse transcriptase kit (Invitrogen, UK). Primers used include alkaline phosphatase (ALP), bone morphogenic protein-2 (BMP-2), vascular endothelial growth factor (VEGF), osteonectin (ON), osteopontin (OP), and osteocalcin (OC). Beta actin was used as an internal control for data normalization. The primers used for the reaction are given in Table 1.

**Table 1:** Primer sequences.

Gene	Sequence
Alkaline phosphatase	FP: TGCAGGATCGGAACGTCAAT RP: GAGTTGGTAAGGCAGGGTCC
Bone Morphogenic Protein-2	FP: CACGAGAATGGACGTGCCC RP: GCAACACTAGAAGACAGCGG
Vascular Endothelial Growth Factor	FP: GCTGCAATGATGAAGCCCTG RP: AAGGCTCACAGTGAATGTGGT
Osteonectin	FP: TGGCCTGTAATAACCCCTGC RP: GGTGTTGCTCTCTCCATCACA
Osteopontin	FP: CCAGCCAAGGACCAACTACA RP: AGTGTTTGCTGTAATGCGCC
Osteocalcin	FP: CTCTTCCCCAGAGTGCAAAG RP: ATTGGTACAGGGAGCACCAG
Beta actin	FP: TGGAGCAAACATCCCCCAAA RP: TGCCGTGGATACTTGGAGTG

### Histology and immunohistochemistry

The parietal calvaria that included the whole defect site (region of interest) was excised and fixed in 10% phosphate-buffered formalin for histological evaluation. The samples were then decalcified using 10% EDTA for two weeks followed by embedding in paraffin blocks. Transverse sections of eight -μm-thickness were made from paraffin blocks using a tissue microtome and stained with hematoxylin-eosin (HE; Sigma, St. Louis, MO).

Immunohistochemical analysis for osteocalcin and osteopontin was performed using anti-rat antibodies. Briefly, the tissue specimens were incubated in a 1:100-diluted anti-osteocalcin antibody solution (Cat no: PA596529) or 1:100 diluted anti-osteopontin antibody solution (Cat no: PA534579) at 37°C for 1 h, followed by 3 washes in phosphate-buffered saline (Sigma, USA). The specimens were further incubated with an anti-rabbit secondary antibody (Abcam, USA). Then, 3,3′-diaminobenzidine tetrahydrochloride (DAB; Dako) was used as a substrate, and the sections were counterstained with Mayer hematoxylin (Dako) and observed under an Olympus BX43 microscope.

### RAMAN spectroscopy

Raman spectroscopic analysis was used to detect the subtle biochemical changes and spectral characteristics in the implanted and sham sites of the rat calvaria. The Raman spectra were collected from RENISHAW inViaTM Confocal Raman Microscope (RENISHAW, UK) with 785 nm edge laser and 0.5% laser power, with 60s and 10s exposure and accumulation time respectively, objective 50L, Renishaw CCD camera detector with a scanning range of 100–3200 cm^-1^.

### Micro-CT imaging

The rat calvaria implantation sites that contained DMB or DCC bone substitutes along with the sham surgery sites were examined by using a Zeiss Xradia 520 Versa, Submicron X-ray Imaging (Carl Zeiss Microscopy GmbH, Germany). All samples were scanned at resolution 36 μm/voxel, voltage 65 Kv and 76 μA power and a fixed threshold of 22000 was selected to segment the bone. A three-dimensional (3D) model was generated and the bilateral critical-sized defect on the rat’s calvarium was visualized and assessed.

After scanning, the resultant images were constructed using Reconstructor Scout-and-Scan 11.1.6411. The density of the experimental implanted defect was determined in Housfield Unit (HU) in the selected circumference by measuring the radiodensity of the bilateral defect along with the implanted bone.

### Statistical analysis

Histomorphometric comparisons between DMB and DCC implanted calvaria sites were performed by non-parametric t-tests. Measurements from micro-CT and gene expression analysis were statistically assessed using two–way ANOVA followed by multiple comparisons using Graph pad prism software (version 9.3.1). P< 0.05 was considered statistically significant. Histomorphometric analyses were performed using IMAGE-J 1.53a (National Institute of Health, Bethesda, MD, USA). Blinded histomorphometric measurements were performed for four histological sections per defect (at ×10 objective magnification). The amount of new bone formed is calculated within the region of interest (ROI) and expressed in percentage.

## Results

A total of 48 Sprague-Dawley rats were successfully operated on and divided into two groups. Group A animals (n = 24) received DMB granules while Group B animals (n = 24) received DCC granules. All animals were active and healthy, gain weight and survived throughout the experimental period.

### Gene expression

**Fig 2:**
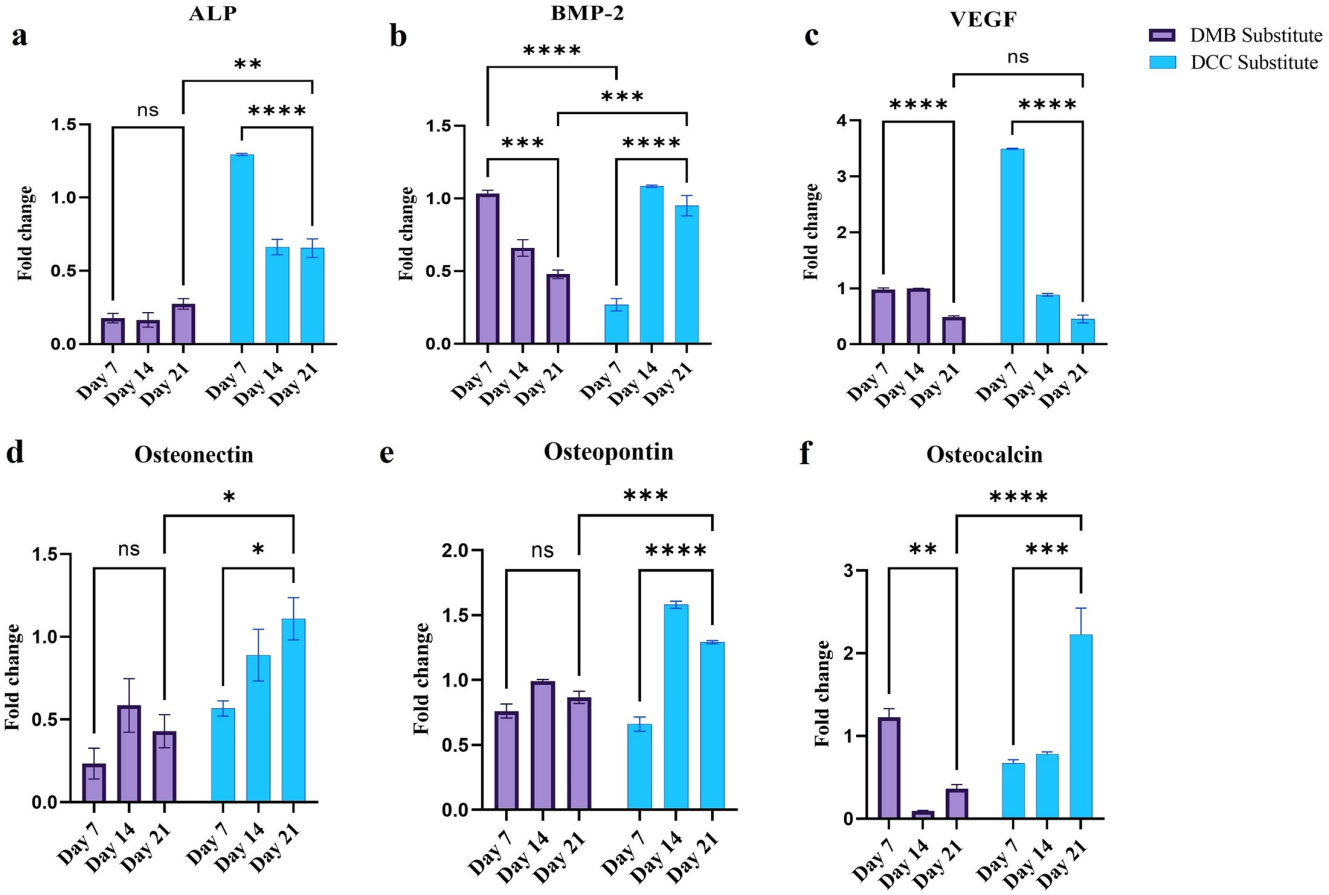
*In-vivo* assessment of osteogenic differentiation markers. Real-time PCR analysis to detect (a) alkaline phosphatase (ALP), (b) bone morphogenic protein-2 (BMP-2), (c) vascular endothelial growth factor (VEGF), (d) osteonectin (ON), (e) osteopontin (OP), and (f) osteocalcin (OC), genes expression at the site of implantation of demineralized (DMB) and decellularized (DCC) bone granules in critical-size calvaria defects.

Significant upregulation of genes associated with osteogenesis such as osteonectin (p<0.05), osteopontin (p<0.0001), and osteocalcin (p<0.001; Fig 2d–2f) was observed by day 21 in DCC substitute implanted sites compared to day 7, whereas the same was not reflected in the DMB sites. Also, a significant upregulation in expression levels of all the osteogenic markers such as ALP (p<0.01), BMP-2 (p<0.001), osteonectin (p<0.001), osteopontin (p<0.001) and osteocalcin (p<0.0001) were significantly higher in the DCC than in the DMB group at day 21 post-surgery. Interestingly, BMP-2 mRNA showed an earlier expression in DMB sites than DCC. BMP-2 mRNA was significantly higher at day 7, compared to DCC sites (p<0.0001; Fig 2b). However, BMP-2 mRNA levels declined significantly by day 21 (p<0.001). By day 21, DCC sites expressed significantly higher BMP-2 mRNA (p<0.001) than DMB.

In the case of vascular endothelial growth factor (VEGF), a high expression was observed at day 7 in both DMB and DCC sites, however its level dropped over time in both the treatment groups. There was no significant difference in VEGF expression detected between DMB and DCC sites by day 21 (Fig 2c). Alkaline phosphatase activity also displayed a similar trend, where their levels were significantly high in the DCC granules implanted sites at day 7 (p <0.0001; day 7 vs day 21; Fig 2a) which was later reduced by day 14 and maintained until day 21. DMB implanted sites demonstrated a lower alkaline phosphatase activity compared to DCC throughout all the time points tested. At day 21, DCC sites expressed higher ALP (p<0.01) than DMB. When the overall gene expression pattern was compared between DMB and DCC groups, DCC demonstrated an earlier and higher expression for all the osteogenic markers.

### Histology

**Fig 3:**
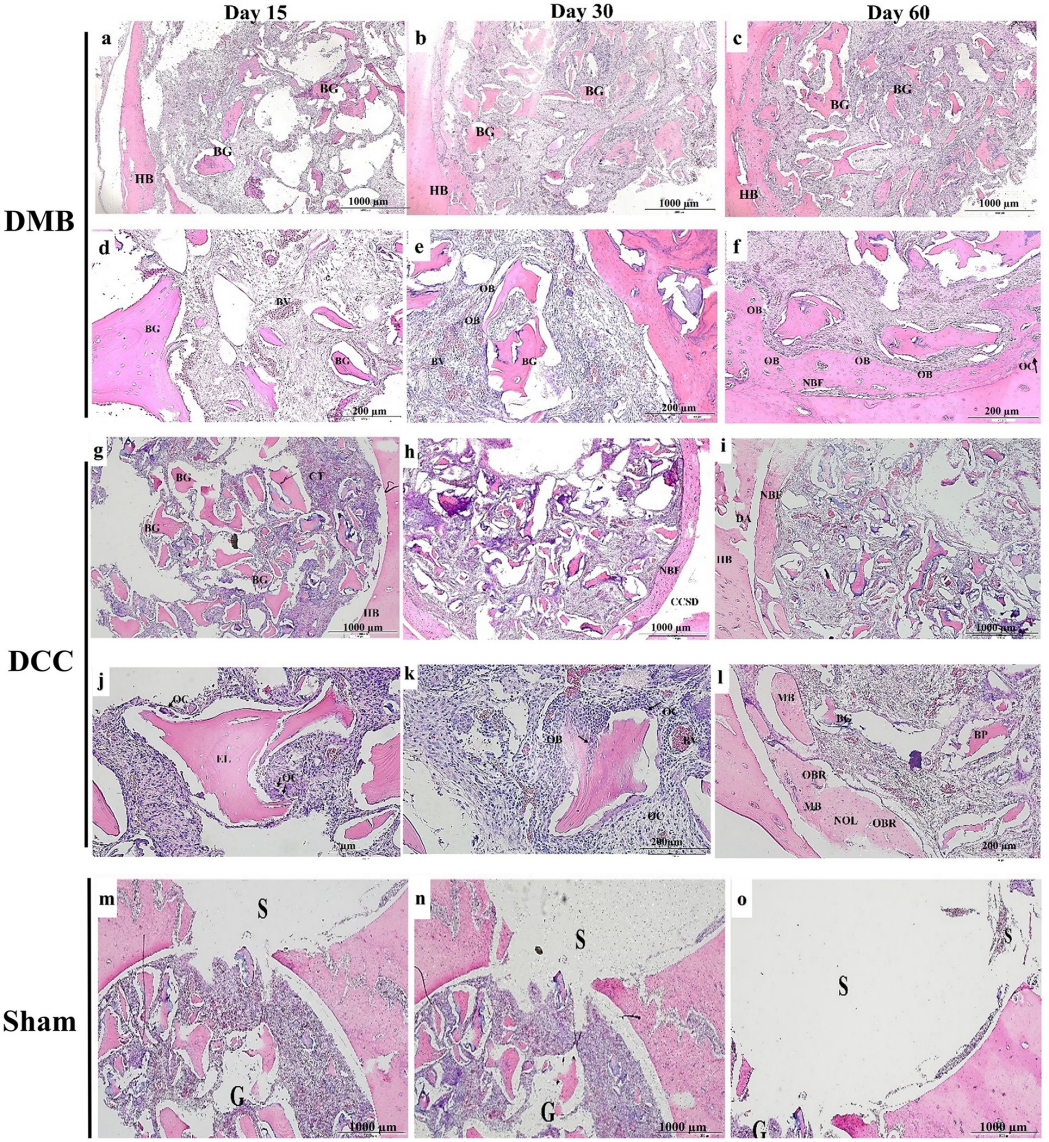
Representative histological features of implantation sites of demineralized bone (DMB) and decellularized (DCC) granules in rat calvaria critical-size defects stained with Hematoxylin and Eosin. DMB (a-f) observed at day 15, 30 and 60 post-surgery. DCC (g-l) observed at day 15, 30 and 60 post-surgery. BG: bone granules; NB: new bone; HB: host bone; BV: blood vessel; OB: osteoblasts; OC: osteoclasts; CT: connective tissue; EL: empty lacunae; MB: mature bone. Hematoxylin and eosin (H&E) staining of DMB and DCC granules implantation sites (G) and sham control site (S) in rat calvaria critical-size defects. (m) H&E staining of the whole bone section showing the DMB implanted area (*right side defect)* and the untreated sham surgery area (*left side defect*) (n) H&E staining of the whole bone section showing the DCC implanted area (*right side defect)* and the untreated sham surgery area (*left side defect*). (o) A representative image of the untreated sham surgery area is shown. During the experimental period, the sham control site remained empty at the periphery of the sham defect.

In the DMB implanted sites, there was gradual and progressive osseous healing demonstrated in the rat calvaria critical-size defects at day 15, 30 and 60 post-surgery, with the treated defects showing superior healing capacity compared to the sham sites (Fig 3). At day 15, the DMB granules displayed empty lacunae in the calvaria defect, interspaced by invading blood vessels in the region of interest (Fig 3a & 3d). Blood vessel formation was more abundant around the implanted DMB granules by day 30 (Fig 3b & 3e) and day 60 (Fig 3c). Also, the newly formed bone was evident around the DMB implanted sites (Fig 3f). On the other hand, there were scanty blood vessels and proliferating fibrous-like tissue in the sham site throughout the 60 days follow-up period.

In the DCC implanted sites, there was also gradual and progressive osseous healing demonstrated in the rat calvaria defects at day 15, 30 and 60 post-surgery, with the treated defects showing superior healing capacity compared to the sham control sites (Fig 3). At day 15, there was a more even distribution of connective tissue fibro-vascular invasion and blood vessels surrounding the DCC granules substitutes (Fig 3g) compared to that of DMB at the same time point. The presence of multinucleated giant cells or osteoclasts-like cells was evident around the implanted DCC granules by day 15 compared to DMB implanted defects (Fig 3j). Blood vessels and osteoblast cells were seen around the periphery of the DCC-implanted bone substitutes by day 30 (Fig 3h & 3k). Osteoclast formation was more readily seen by day 30 (Fig 3k) around the periphery of the DCC granules. By day 60, new bone formation was seen (Fig 3i & 3l). The new mature bone formed was seen migrating from the periphery towards the center of the defect and some islands of new bone formation were separated from the host bone (Fig 3l). It is interesting to note that there were more new mature bones seen histologically in DCC granules implantation sites (Fig 3l) compared to DMB implantation sites at day 60 (Fig 3f). During this healing period, the sham control site remained empty and did not show any evidence of new bone formation in both group of animals (Fig 3m–3o).

### Histomorphometric analysis

**Fig 4:**
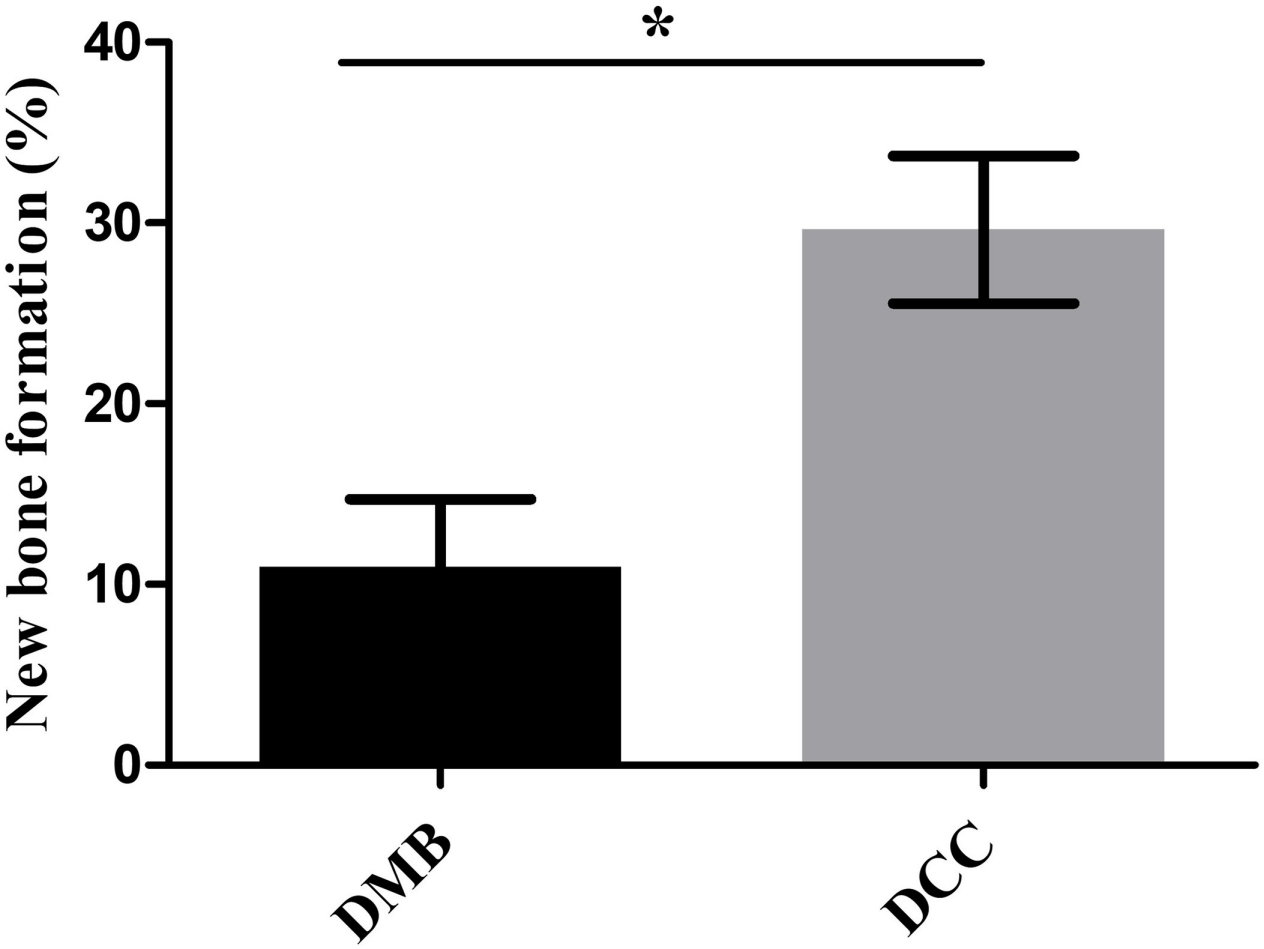
Quantification of histomorphometric analysis of new bone formation at DMB and DCC granules implanted into calvaria critical-size defects sites. The area of new bone formation was quantified in percentage using Image J software, p<0.05.

New bone formation was evident in the histological and histomorphometric assessment of both DMB and DCC granules implanted sites in the margin of the defects. However, when compared to DMB implanted sites, a significant increase in new bone formation was observed in the DCC sites (Fig 4; p<0.05) with the presence of fibrous tissue around the remaining granules in the central area.

### Immunohistochemical analysis

**Fig 5:**
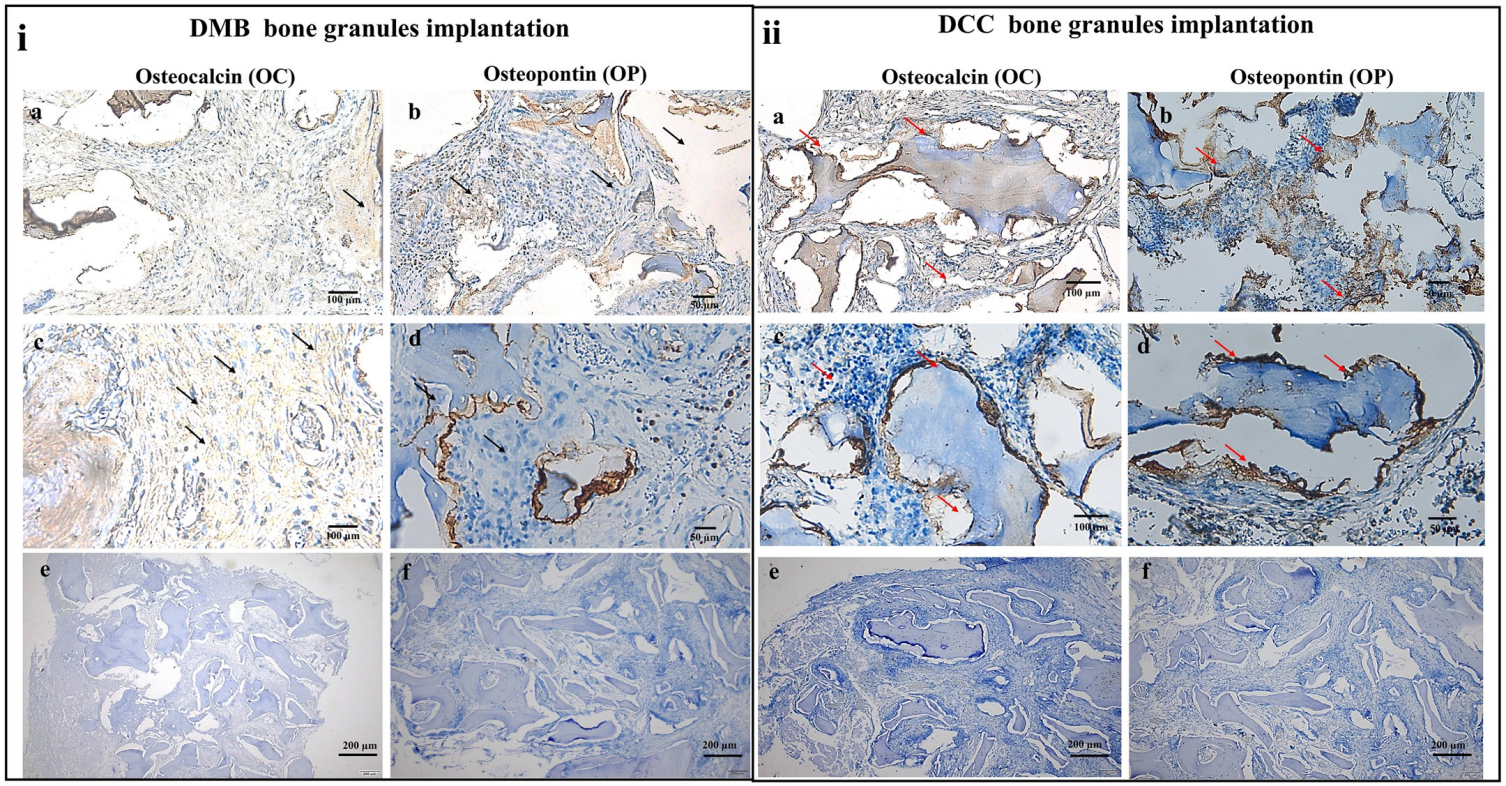
Representative images of immunohistochemical staining of DMB and DCC implantation sites to visualize the intensity distribution of osteogenic factors. (i) Osteocalcin (a and c) and osteopontin (b and d) in calvaria defects at day 60 post-implantation of DMB granules at low (a and b) and high (c and d) magnification respectively. The positive signal associated with the new bone matrix appears as brown staining (arrows), both around and between implanted DMB granules. Osteocalcin expression is evident at the edges of the defect and in the new bone matrix bridging the DMB granules. Histochemical staining for osteopontin (b and d) exhibited deposition at the boundaries and within the tissue, suggesting the presence of active osteoblast cells in the bone remodeling process. (ii) Osteocalcin (a and c) and osteopontin (b and d) in calvaria defects at day 60 post-implantation of DCC granules at low (a and b) and high (c and d) magnifications respectively. A stronger positive signal associated with the new bone matrix appears as brown staining (red arrows), both around and between implanted DCC granules. Osteocalcin expression is evident at the edges of the defect and in the new bone matrix bridging the DCC granules. Immunohistochemical staining for osteopontin (b and d) exhibited strong deposition at the boundaries of the degrading DCC granules and throughout the newly formed bone tissue, suggesting the presence of active osteoblast cells in the bone remodeling process. Negative control images for the respective stains are shown in the lower panel (e-f).

Immunohistochemical staining for osteocalcin in the DMB granules implanted sites showed a vague intensity on day 15 and a slightly clearer staining by day 30. Similarly, the staining for osteopontin remained vague on days 15 and 30. By day 60 post-surgery, DMB implanted sites showed clearer and more distinct staining for both the osteocalcin (i a & c) and osteopontin (i b & d) proteins, indicating active bone regeneration at the periphery and extending towards the center of the critical-sized defect (Fig 5i).

Immunohistochemical staining for osteocalcin marker in the DCC granules implanted sites showed a generally similar feature to DMB whereby low-intensity staining was observed both on day 15 and 30, while a more evenly distributed and stronger positive staining was demonstrated at day 60 (Fig 5ii). Immunohistochemical staining for osteopontin also showed similar vague staining intensity at days 15 and 30. By day 60, positive staining for both osteogenic markers, osteocalcin (Fig 5iia & 5iic) and osetopontin (Fig 5iib & 5iid) were observed around the DCC granules and extending towards the periphery. Immunohistochemical staining for osteopontin marker showed strong deposition at the boundaries of the degrading DCC granules and within the newly formed bone tissue, suggesting the presence of active osteoblast cells in the bone remodeling process.

### Micro-CT evaluation of the calvaria implantation sites

**Fig 6:**
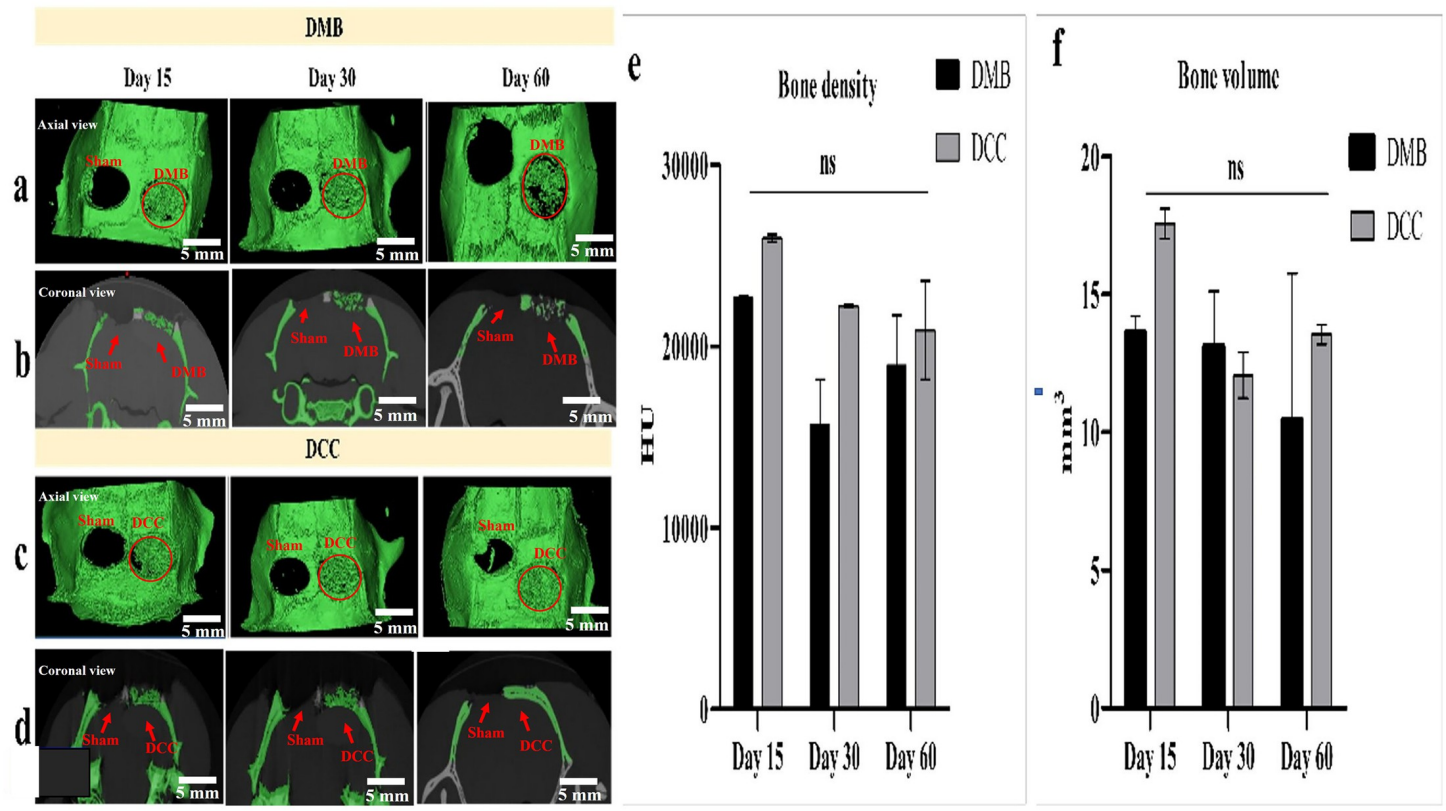
Qualitative and quantitative Micro-CT analysis of the calvaria critical-size defects implanted with demineralized (DMB) and decellularized (DCC) bone granules. Representatives of (a) axial (b) coronal view of DMB implanted sites; and (c) axial (d) coronal view of DCC implanted sites of the calvaria defects. (e) Quantification of bone density at DMB and DCC granules implantation sites at day 15, 30 and 60 post-implantations, analyzed using micro-CT. No significant difference in bone density was observed between DMB and DCC sites throughout all time points. (f) Quantification of bone volume at DMB and DCC granules implantation sites at day 15, 30 and 60 post-implantations, analyzed using micro-CT. No significant difference in bone volume was observed between DMB and DCC sites throughout all time points.

Micro-CT images of the implanted sites of DMB and DCC granules indicated new bone formation at the sites of the calvaria defects by progressive replacement of the implanted granules through resorption and formation of new bone in axial view (Fig 6a and 6c). The closure of the bone defect in the DCC implanted group was observed in the coronal view of the micro-CT image (Fig 6d), in contrast to the DMB group where the bony healing is discontinuous and incomplete (Fig 6b), providing a clear difference in the efficacy of the healing process between both groups. Micro-CT analysis further showed a trend of lower bone density at DMB implanted sites compared to DCC, however, the observed difference was not statistically significant (Fig 6e). Similarly, the bone volume was consistently lower in DMB compared to DCC at all three-time points. In the case of the DCC group, the bone volume trend initially decreased from day 15 to day 30, but an upward trend indicating an increase in volume was demonstrated by day 60 (Fig 6f).

### Raman spectra analysis

**Fig 7:**
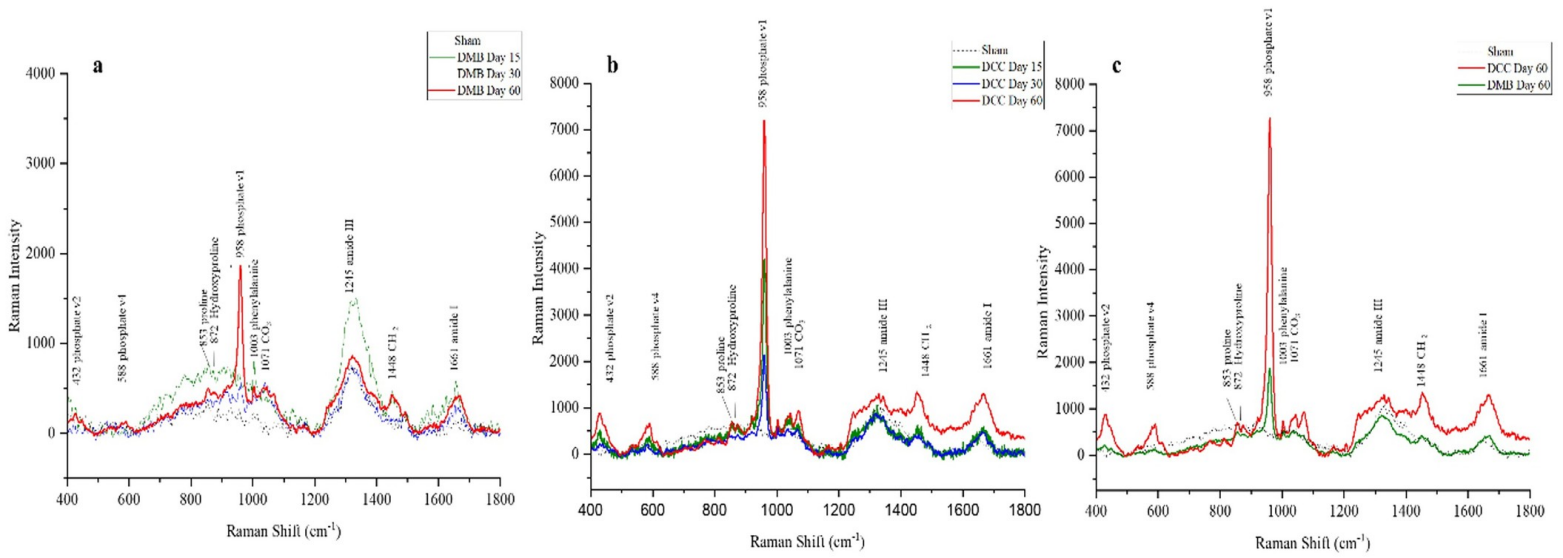
Raman spectroscopy analysis of the DMB and DCC implanted calvaria defects. (a) Normalized RAMAN spectra were acquired from post-implantation sites of DMB granules at days 15, 30 and 60. (b) Normalized RAMAN spectra were acquired from post-implantation sites of DCC granules at days 15, 30 and 60. (c) Combined Normalized RAMAN spectra acquired from post-implantation sites of DMB and DCC granules at day 60 are shown.

Fig 7 shows the normalized Raman spectra (averaged across study groups) acquired from DMB and DCC implanted sites and compared to sham control sites at day 15, 30 and 60 post-implantations. Bone tissue spectra obtained by Raman spectroscopy were in the spectral range of 400 to 1800 cm^−1^. The collagen-dominated protein identified by proline at 919 cm^-1^ signifies the resorbable matrix factor while carbonated apatite identified by PO4 ^3-^ at 958 cm^-1^ and CO_3_^2-^ at 1072 cm^-1^ signifies the resorbable mineral factor at both the DMB and DCC granules implanted sites (Fig 7a & 7b). The Raman spectrum at the DMB and DCC implanted sites showed peaks at 1065–1070 cm^−1^, 945–964 cm^−1^, and 919 cm^−1^, as previously reported for native bone. The spectral peak for PO4 ^3-^ was highest at day 60 for both DMB and DCC implanted sites (Fig 7a & 7b). In addition, the collagen bands seen at specific wave numbers such as 853, 872, 1242–1340, 1446, and 1660–1690 cm^-1^ corresponds to proline, hydroxyproline, amide III, proteins CH2 deformation and amide I band respectively in the DMB and DCC implanted calvaria defects over the different time points. At day 60, the spectral peak for PO4 ^3-^ was highest for DCC implanted site than DMB (Fig 7c).

## Discussion

Aggressive decellularization techniques damage the delicate extracellular matrix and its regenerative cues and the efficacy of a decellularized bone substitute becomes questionable. Thus, the two main diverse concepts of producing a natural bone scaffold are whether to protect the extracellular matrix while dissolving the mineral content, as such producing the DMB or to keep the mineral content and decellularize the organic cellular components, as such producing the DCC in this study. Interestingly both types of bone grafts have demonstrated different clinical results in the healing of rat critical-size defect model.

Although there are many reported in-vivo and clinical studies examining the use of bovine bone grafts for bone regeneration, few publications are evaluating the bone regeneration potential of decellularized bovine bone grafts [8]. The aim of this study was to assess the osteogenic potential of DCC granules, and compared them to DMB granules, using gene expression analysis, histological assessment, micro-CT, and RAMAN spectroscopy in supporting the closure of rat calvaria critical-size defect model over an 8-week healing period.

The development and characterization of DCC bovine bone granules used in this study are detailed in a recent publication from our group and this scaffold has shown its potential to promote osteogenic and osteoconductive properties through *in vitro* experiments [13]. The positive results obtained from this *in vitro* study are subsequently corroborated in the present *in vivo* study through a range of experiments, including histology, histomorphometry, immunohistochemistry, micro-CT, gene expression and RAMAN spectra analysis.

The size of the bone graft granule is a significant determinant of bone healing responses [21]. It has a significant effect on bone formation, mineralization and bone mineral density at the defect zone [22]. According to Leiblein *et al*., granules with sizes between 0.5 and 1.0 mm demonstrated noticeably better bone repair than larger granule sizes [21]. Hence, in the current study, the DMB and DCC granules used were in the range of between 0.5 mm and 0.75 mm in size for bone defect regeneration which was confirmed by the SEM analysis.

Markers of osteoblast function such as ALP, BMP-2, ON, OC, OP, and the proangiogenic factor VEGF were also assessed by gene expression studies. The levels of ALP peaked in the first phase of bone healing in the DCC group on day 7 and later declined by day 14 and remained low until day 21. During the osteogenic differentiation of MSCs, the levels of ALP typically reach their peak in the initial phase of mineralization, usually within the first week. Subsequently, as mineralization and calcium formation are induced, the ALP activity starts to decline [23]. Therefore, the significant decrease in ALP activity observed in the DCC group, accompanied by an increase in mineralization, reflects a typical expression profile widely described in osteogenic differentiation studies [24]. ALP expression is highly prominent in the starting phase of the mineralization [25], but undergoes a downregulation as mineralization progresses. In contrast to this, the DMB group displayed a minimal increase in ALP levels from day 7 to 21, although not significant, but the level is much lesser than DCC at all time points.

The DCC group demonstrated an up-regulation of BMP-2 mRNA level, which is known to be a crucial factor in facilitating new bone growth. BMPs play a crucial role in the growth and differentiation of osteoblastic lineage cells. They have demonstrated potent abilities to stimulate bone formation in various animal models [26]. The significantly upregulated of BMP-2 as seen in the current study on day 21 in DCC further indicated the plausible occurrence of a prompt and robust level of bone regeneration under the induction of BMP-2 at the DCC implanted sites. In contrast, a decline in BMP-2 levels was observed at the DMB sites. The demineralization process during the production of DMB granules could have contributed to the earlier release of BMP-2 from the surface of the bone, which subsequently diminished over time [27, 28]. This could explain the initially elevated mRNA levels for BMP-2 at day 7 in the DMB group, followed by a decline at day 14 and day 21.

On examining the gene expression of VEGF in this study, we found that the level was highly upregulated at day 7 in the DCC group alone which later significantly reduced by day 14 and further downregulated by day 21. This is in agreement with a previous study by Shinji *et al*. in 2005, in a rat model of bone regeneration that observed peak expressions of VEGF during the first week [29]. The initial phase of bone healing and new bone formation also requires the adequate formation of new capillaries from existing blood vessels by the process of angiogenesis [29]. In addition to restoring blood flow to the injured site, which is essential for the healing process, angiogenesis facilitates cellular communication and signaling among different cell types involved in bone healing [30]. Osteoprogenitor cells, which differentiate into bone-forming cells called osteoblasts, migrate along the newly formed blood vessels to the injury site, contributing to the formation of new bone tissue [31].

ON is one of the main non-collagenous proteins of bone tissue that are expressed by osteoblasts and some osteocytes. In the present study, we noted a progressive rise in ON expression throughout all the time points, with the highest level observed by day 21 in DCC implanted sites, indicating active osteogenic activity. While a similar pattern was observed in the DMB implanted sites, the expression levels were significantly lower compared to the DCC group.

The intense staining for OC in the DCC implanted calvaria sites on day 60 suggests that the DCC group is at a more advanced stage in the bone remodeling process compared to the DMB group. The data is consistent with the gene expression studies where an enhanced expression for OC is observed for the DCC group at all the time points in comparison to the DMB group. The intense expression of OP as observed in the current study further suggested the active bone remodeling and tissue regeneration processes occurring in both the DMB and DCC implanted sites. Although OP expression was lower at the mRNA level in the DMB group than in the DCC, OP expression in the DMB and DCC groups was comparable by immunohistochemical analysis. During the process of osteoblast differentiation and mineralization, OC and OP are synthesized and released. OP is an early active marker of bone formation and OC expression is reported in the later phase of bone formation [32]. The presence of OC and OP in the ECM plays a significant role in various aspects of bone metabolism, indicating osteoblast differentiation, mineralization and cell attachment [33]. Hence their presence is crucial for promoting new bone formation and integration with the surrounding tissue. The gene expression analysis which was supported by immunohistochemical staining in this study further suggests that DCC granules represent a biomimetic scaffold that was able to support osteogenesis and new bone formation at a faster pace in comparison to the DMB group.

Histological and Micro-CT analysis revealed the ability of the granules to resorb and replace by new tissue during the healing period. This critical characteristic was confirmed by changes in the density and volume of both DMB and DCC in Micro-CT analysis. Caplanis *et al*. did not find any histological effects on bone formation in canine alveolar defects treated with demineralized freeze-dried bone allografts for up to 4 weeks [34]. Similarly, in a previous study using demineralized freeze-dried bone for repair of rat calvaria models, Intini et. al found no effective bone formation was observed for up to 8 weeks of follow-up [35].

In the current study, both DMB and DCC granules displayed a considerable degree of new bone formation along the margins of the defect as confirmed by histological, histomorphometric and immunohistochemical analysis. The presence of fibrous tissue around the remaining granules in the central area was also seen in both DMB and DCC implanted sites. This finding is consistent with previous studies that have reported similar observations when using bone grafts from alternative natural sources, such as porcine [8]. The fibrovascular invasion with blood vessels around the bony granules allowed new bone formation in a centripetal manner which began from the margins of the defect. The newly formed bone at the DMB and DCC implanted sites further showed incorporation with the host bone as reported in similar studies previously [36]. However, compared to DMB, DCC sites exhibited a higher amount of new bone formation, characterized by the presence of mature bone in the healing defect site. The histological features of the DCC group displayed a greater abundance of mature bone and the presence of a more prominent osteoblastic rim in comparison to the DMB group. Additionally, in the DCC group, a few islands of newly formed bone appeared in the surrounding area of the defect, suggesting a more advanced stage of de novo bone healing. On the other hand, in the DMB group, the newly formed bone largely remained connected to the peripheral host defect, suggesting a relatively less mature stage of bone development.

Further, in our study, RAMAN spectroscopy [37] was conducted to detect the mineral and protein composition at the defect sites over the various time points post-implantation with DMB and DCC granules. The spectra of the defects showed differences concerning mineral and collagen content between the sites implanted with DMB or DCC granules. The RAMAN spectroscopy analysis revealed a highly significant increase in phosphate levels by day 60 in defects implanted with DCC bone granules supporting the increased new bone formation observed by histomorphometry. Although immunohistochemistry showed comparable osteogenic marker intensities between DMB and DCC, the histomorphometric analysis clearly showed more abundant bone regeneration in the DCC granules implanted sites, supported by higher osteogenic marker activity and peak mineral and collagen content demonstrated by RAMAN spectroscopy. Islands of new bone formation were observed in the DCC healing sites suggesting the presence of de novo bone formation and osteoinductive activity. The comprehensive observations incorporating histological and immunohistochemistry examination, gene expression, and RAMAN spectroscopy, collectively suggested the superior osteoconductive, osteoinductive and osteogenic properties of the DCC granules when compared to the DMB granules in this study. These findings also provide evidence for the enhanced support for bone-regenerating capabilities of the DCC group, supporting its potential as a promising option for bone graft substitutes.

Micro-CT images analysis in axial and coronal views showed defects implanted with DCC granules formed a condensed fused scaffold united with the host bone while the DMB implanted sites showed empty defects. The progressive bone regeneration shown in the micro-CT analysis began with islands of new bone on day 15 which is not quite connected with the native bone, while on day 30, the islands appeared denser and bridged together. By day 60, the newly formed calvaria bone generated an outer and inner cortex in continuity with the native host bone. The bone density and bone volume were also found to be higher in the DCC compared to DMB implanted sites. In the untreated sham surgery sites, there was no evidence of active bone regeneration shown on day 60, however, there were incomplete bone islands and fibrous tissue observed by day 60.

In conclusion, this study supported the growing body of evidence that suggest decellularized bone scaffold enhances osteogenic performance in bone regeneration compared to demineralized bone scaffolds, though the underlying molecular mechanisms remain to be fully elucidated. The low calcium and phosphorous content in Demineralized Bone Matrix has been hypothesized as one of the contributing factors towards its less favorable osteogenic property.

Shih et al. (2014) demonstrated a molecular mechanism through which CaP minerals induce osteogenesis of human mesenchymal stem cells with an emphasis on phosphate metabolism [38]. Our study supported their findings regarding the need to preserve calcium phosphate (CaP) moieties during decellularization process of bovine bone graft. This protects the extracellular matrix scaffold mineral environment that facilitate osteogenicity and osteoinductivity for treating critical bone defects. Our DCC granule produced in this study was able to promote healing in rat calvaria critical-size defect by supporting osteoprogenitor cells adhesion, proliferation and differentiation, with subsequent mineralization of extracellular matrix. The DCC implanted sites demonstrated features of remodeling characterized by bone degradation and formation occurring in the microenvironment, with the classical scaffold properties of osteoconduction, osteoinduction and osteogenesis.

## Supporting information

S1 File (PDF)
