# Correction: Evaluation of the osteogenic potential of demineralized and decellularized bovine bone granules following implantation in rat calvaria critical-size defect model

**DOI:** 10.1371/journal.pone.0298012

**Published:** 2024-01-25

**Authors:** Ali Al Qabbani, K. G. Aghila Rani, Sausan AlKawas, Suzina Sheikh Abdul Hamid, Johari Yap Abdullah, A. R. Samsudin, Ahmad Azlina

The fifth author’s name is spelled incorrectly. The correct name is: Johari Yap Abdullah. The correct citation is: Al Qabbani A, Rani KGA, AlKawas S, Sheikh Abdul Hamid S, Yap Abdullah J, Samsudin AR, et al. (2023) Evaluation of the osteogenic potential of demineralized and decellularized bovine bone granules following implantation in rat calvaria critical-size defect model. PLoS ONE 18(12): e0294291. https://doi.org/10.1371/journal.pone.0294291
